# Selective crack suppression during deformation in metal films on polymer substrates using electron beam irradiation

**DOI:** 10.1038/s41467-019-12451-8

**Published:** 2019-10-01

**Authors:** So-Yeon Lee, Kyung Ryoul Park, Sung-gyu Kang, Ji-Hoon Lee, Eun-chae Jeon, Cheol-Hwee Shim, Jae-Pyoung Ahn, Dong-Ik Kim, Heung Nam Han, Young-Chang Joo, Changsoon Kim, In-Suk Choi

**Affiliations:** 1 Department of Materials Science and Engineering, Seoul National University, 1 Gwanak-ro, Gwanak-gu, Seoul, 08826 Republic of Korea; 2 Graduate School of Convergence Science and Technology, and Inter-University Semiconductor Research Center, Seoul National University, 1 Gwanak-ro, Gwanak-gu, Seoul, 08826 Republic of Korea; 3 Materials Center for Energy Convergence, Surface Technology Division, Korea Institute of Materials Science (KIMS), 797 Changwon-daero, Seongsan-gu, Changwon, Gyeongnam 51508 Republic of Korea; 4 School of Materials Science and Engineering, University of Ulsan, 93 Daehak-ro, Nam-gu, Ulsan, 44610 Republic of Korea; 5 Advanced Analysis Center, Korea Institute of Science and Technology, 5 Hwarang-ro 14-gil, Seongbuk-gu, Seoul, 02792 Republic of Korea; 6 Center for Energy Materials Research, Korea Institute of Science and Technology, 5 Hwarang-ro 14-gil, Seongbuk-gu, Seoul, 02792 Republic of Korea; 7 Research Institute of Advanced Materials (RIAM), Seoul National University, 1 Gwanak-ro, Gwanak-gu, Seoul, 08826 Republic of Korea

**Keywords:** Surfaces, interfaces and thin films, Electronic devices, Composites, Mechanical properties

## Abstract

While cracks are usually considered detrimental, crack generation can be harnessed for various applications, for example in ceramic materials, via directing crack propagation and crack opening. Here, we find that electron beam irradiation prompts a crack suppression phenomenon in a copper (Cu) thin film on a polyimide substrate, allowing for the control of crack formation in terms of both location and shape. Under tensile strain, cracks form on the unirradiated region of the Cu film whereas cracks are prevented on the irradiated region. We attribute this to the enhancement of the adhesion at the Cu–polyimide interface by electrons transmitted through the Cu film. Finally, we selectively form conductive regions in a Cu film on a polyimide substrate under tension and fabricate a strain-responsive organic light-emitting device.

## Abstract (web summary)

Cracking in thin films is usually associated with failure. Here, the authors use an electron beam to selectively irradiate a copper thin film on a polymer substrate and introduce designed cracks under tension to make a strain responsive organic light-emitting device (OLED).

## Introduction

Cracks should be avoided unless we can control them. Crack formation has been considered a fatal failure, and most studies on cracks have focused on suppressing crack generation. However, researchers have recently attempted to control crack formation and even utilize the beneficial effects of cracking^1–5^. For instance, cracks in thin ceramic materials can be controlled in paths with widths down to sub-nanometers by residual stress engineering, which can be used as nanofluid channels for lab-on-a-chip applications^6,7^. While most of the studies on crack control are for ceramic thin films, some studies have proposed techniques to suppress crack formation in metallic thin films on polymer substrates by enhancing the adhesion between the film and the substrate because major electrical circuit failure in flexible and stretchable electronics occurs at metallic interconnects or current collectors that are susceptible to cracking due to frequent and severe deformation such as bending or stretching^8–11^. However, the proposed processes are hardly applicable to submicron-thick metallic films because of the brittle nature of the adhesive interlayers and rough surfaces caused by surface modification^12,13^. Moreover, controlling the shape and area of crack formation in thin metallic films is even more challenging compared to ceramic thin films because the general crack formation behavior of metallic films is very different from the crack formation behavior of ceramic thin films due to completely different fracture mechanisms arising from plastic deformation in metals^10^. Consequently, possible applications of selective crack formation in metallic thin films have been beyond the conventional research scope.

In this paper, we introduce a method for controlling the crack formation in a metallic thin film on a polymer substrate by utilizing unique electron beam–matter interactions at the nanoscale. We found that when the electron beam (e-beam) irradiates the surface of a 100-nm-thick Cu film deposited on a polyimide (PI) substrate, the crack formation is significantly suppressed so that the e-beam-irradiated area is hardly cracked, even at a tensile strain of 30%. The controlled e-beam patterns can even generate a non-crack pattern of any shape in the metallic thin film upon applying a tensile load. Experiments and simulations have shown that transmitted electrons can alter and engineer the interface between the Cu thin film and the PI substrate, leading to the improved adhesion between the Cu thin film and the PI substrate, thereby suppressing crack formation during tensile deformation. By utilizing e-beam irradiation, we can not only achieve selective suppression of crack formation but also introduce a strain-responsive conductivity pattern in the metallic thin film because of the difference in electrical conductivity between the cracked and non-cracked areas. Hence, we further incorporate our non-destructive and mask-free crack patterning method into the fabrication of an organic light-emitting device (OLED). We have successfully fabricated a phosphorescent OLED on a Cu thin film with an e-beam irradiated pattern, which results in a strain-induced light emission pattern when a tensile load is applied. We believe that our technology can be applicable not only to enhancing crack resistance in flexible and stretchable devices but also to developing smart devices engineered by strain-responsive conductive patterning.

## Results

### Crack suppression in a metal film using e-beam irradiation

**Fig. 1:**
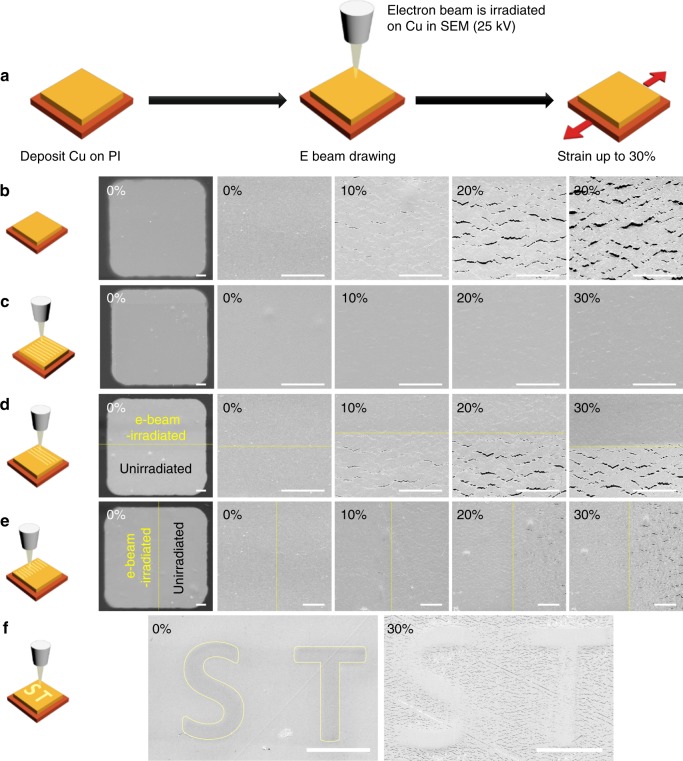
Effect of electron beam (e-beam) irradiation on the crack behavior of Cu thin films during tensile loading. **a** Schematic diagram of e-beam irradiation and subsequent tensile testing. SEM images of Cu thin films deposited on polyimide (PI) substrates were captured at various levels of tensile strain, 0, 10, 20, and 30% (**b**) without irradiation and (**c**) with irradiation on the entire Cu film. The e-beam irradiation effectively suppresses the crack formation in the irradiated region even at a high tensile strain. SEM images of the Cu thin films irradiated only in specific region(s) such as (**d**) the upper half region, (**e**) the left half region, and (**f**) in ‘S’-shaped and ‘T’-shaped regions are shown. The local e-beam irradiation selectively prevents the crack formation in the Cu thin films on PI substrates. Scale bars, 10 μm (**b**–**e**); 100 μm (**f**)

Figure 1a describes our experimental procedure to investigate the effect of e-beam irradiation on the crack behavior of a Cu thin film under tensile deformation. Briefly, samples, each consisting of an array of 100-nm-thick rectangular (100 × 100 µm) Cu thin film on a 125-µm-thick PI substrate, were exposed to e-beam irradiation with an acceleration voltage (*V*_A_) of 25 kV in a scanning electron microscope (SEM) and subsequently observed under in situ tensile testing with gradually increasing tensile strain (*ε*) oriented along the vertical direction in Fig. 1b to f. More detailed description of the experiment is described in the Methods section, Supplementary Fig. 1, and Supplementary Table 1. Figure 1b shows the result of a control experiment (i.e., an experiment without e-beam irradiation). At *ε* = 10%, which is a pre-crack stage, a morphological change in a form of wavy lines can be seen, with microcrack nucleation observed in some areas. As *ε* increases, the number of microcracks increases, and the microcracks are then merged via crack propagation along a direction perpendicular to the tensile direction, resulting in cracks distributed over the entire film at *ε* = 20%. Further increase in *ε* leads to the Cu thin film delaminated at the crack peripheries, as shown in the rightmost image in Fig. 1b (*ε* = 30%). This type of crack behavior during initial tensile deformation is a phenomenon commonly observed for Cu thin films deposited on flexible substrates and has been reported in many previous studies^8–10^. In contrast, the e-beam irradiation is found to remarkably suppress the crack generation under tensile deformation. When the e-beam has irradiated the entire area of the Cu thin film with a beam current (*I*) of 11 nA and a dose (*D*) of 4.52 × 10^4^ µC cm^−2^ (Fig. 1c), the Cu film is found to be crack-free at *ε* = 10%, and even at *ε* = 20%, it is almost crack-free, where only several microcracks of very small sizes are observed. When *ε* reaches 30%, microcracks with sizes comparable to those found in the unirradiated film at *ε* = 10% (Fig. 1b) are formed (more detailed comparison of crack behavior of the Cu thin films can be found in the in situ video provided in Supplementary Video 1). It should be noted that the conventional Cr adhesion layers are hardly applicable to suppress crack formation by adhesion enhancement for submicron-thick Cu films because of the brittle nature of the adhesive interlayers initiating crack formation in nano-metallic thin films^12^. Our experiment also showed the same crack formation in the Cu thin film with a Cr adhesion layer as shown in Supplementary Fig. 2.

The crack suppression by e-beam irradiation is more evident when the e-beam has irradiated only specific region(s) in the Cu thin films. For example, when only the upper half region of the Cu thin film is irradiated with the e-beam (*I* = 11 nA, *D* = 4.52 × 10^4^ µC cm^−2^), that region remains crack-free at *ε* = 30%, while in the unirradiated lower half region many cracks are formed (Fig. 1d), with a degree of cracking similar to that of the Cu thin film whose entire area is unirradiated (rightmost image of Fig. 1b). Likewise, as shown in Fig. 1e, the e-beam irradiation in the left half region of the Cu thin film effectively suppresses the crack formation in that region (*I* = 11 nA, *D* = 4.52 × 10^4^ µC cm^−2^). In addition, in this case, propagation of cracks formed in the unirradiated right half region stops at the boundary of the two regions, indicating that the e-beam irradiation can also prevent the crack propagation due to mode I fracture that occurs in the samples shown in Fig. 1b, d. Furthermore, the suppression of formation and propagation of cracks by e-beam is found to be equally effective when the boundaries of the irradiated regions have an arbitrary orientation with respect to the tensile direction. As shown in Fig. 1f, at *ε* = 30%, cracks are not generated only in the ‘S’-shaped and ‘T’-shaped regions that underwent the e-beam irradiation (*I* = 45 nA*, D* = 9.02 × 10^4^ µC cm^−2^), whose boundaries are marked by yellow lines. Our results indicate that local e-beam irradiation followed by stretching allows for selective prevention of crack formation in the Cu thin films on the PI substrates  (Cu film-PI system).

### Analyses of e-beam induced changes in the Cu film–PI system

**Fig. 2:**
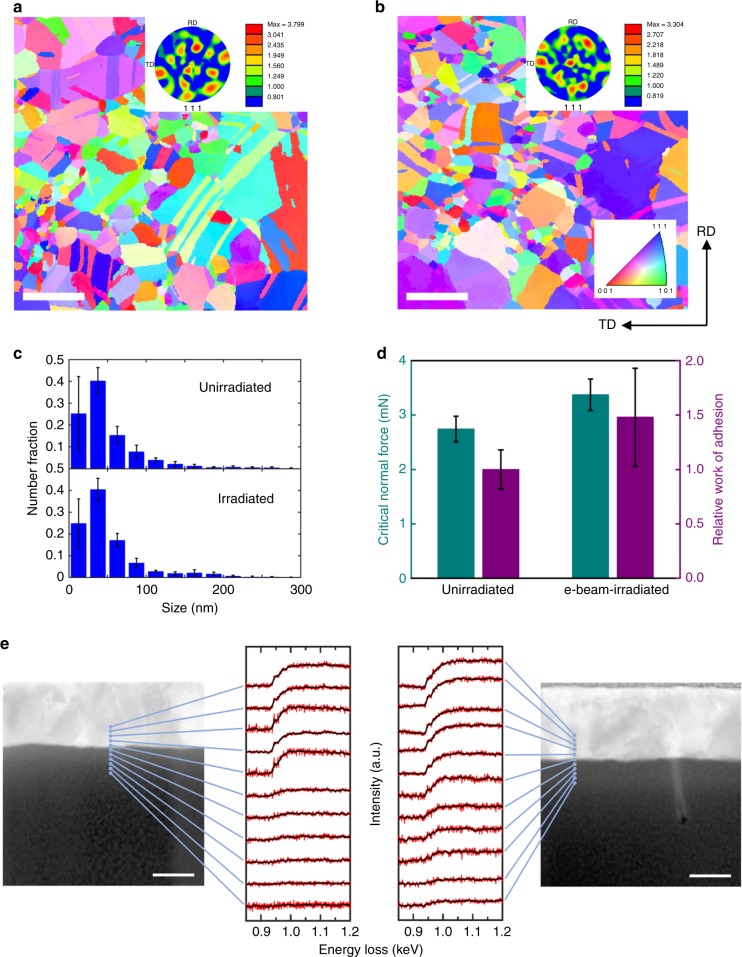
Investigation of e-beam induced changes in the Cu film–PI system. Inverse pole figure maps and the inset of (111) pole figures of (**a**) unirradiated and (**b**) e-beam-irradiated Cu thin films obtained from ASTAR^TM^ analysis and (**c**) the grain-size distribution in each case. **d** The critical normal force (*L*_cr_) extracted from a nanoscratch test measuring the normal force at the Cu–PI interface at the onset of delamination. The relative value of work of adhesion (*W*) between irradiated and unirradiated samples was then calculated, showing better adhesion for the irradiated sample. **e** The electron energy loss spectroscopy data collected from different positions at 5 nm intervals across the Cu–PI interface for the unirradiated (left) and irradiated (right) samples. Red lines are the raw data, and black lines are their averages over 20 neighboring points. The probe positions are denoted on the high-angle annular dark field scanning transmission electron microscopy images. Error bars represent the standard deviation (**c**, **d**). Scale bars, 200 nm (**a**, **b**); 50 nm (**e**)

When a solid material is irradiated with electrons, they can be transmitted or reflected by elastic collisions. Alternatively, by inelastic collisions, the energy of the electrons can be absorbed in the material, which often results in the change in material properties^14^. Therefore, it is necessary to investigate whether the e-beam caused a structural change in the Cu film, which can suppress the crack formation by enhancing its ductility. Figure 2a, b show the representative textures of the unirradiated and irradiated Cu thin films, respectively, which were obtained using the recently developed automatic crystal orientation and phase mapping package in a scanning transmission electron microscope (STEM), referred to as ASTAR™. The e-beam irradiation condition was *V*_A_ = 25 kV, *I* = 11 nA, and *D* = 4.52 × 10^4^ µC cm^−2^, which was also the case for other irradiated samples in Fig. 2. In both cases, the Cu thin films consist of nanocrystals in various sizes, with nanotwins present in most of them. The inverse pole figure maps and (111) pole figures of the Cu thin films shown in Fig. 2a, b and the insets indicate that the texture of the as-deposited Cu thin film remains random without an evolution of a specific texture after the e-beam irradiation. In Fig. 2c, the grain size distributions of the unirradiated and irradiated Cu thin films are quantitatively compared, showing that the two distributions are very similar: in both cases, most grains are smaller than 100 nm in size, and the number fraction peaks at an identical size range (25–50 nm); there is almost no difference in average grain size (54.8 ± 13.0 nm and 53.4 ± 5.7 nm for the Cu thin film without and with the electron irradiation, respectively). In addition, the lengths of low-energy and twin boundaries, which play an important role in increasing the ductility of nanocrystalline Cu, show almost no change upon the e-beam irradiation. The fraction of the length of high-energy boundaries was slightly increased from 0.42 to 0.46 due to the e-beam irradiation, but the overall length distribution was unaltered, and no growth or disappearance of specific misorientations is observed (Supplementary Fig. 3). These results indicate that the electron energy transferred to the Cu film in our case is sufficiently low that the nanostructure of the Cu film remains unaltered. This is not surprising because the e-beam irradiation condition in our case (*V*_A_ ≤ 25 kV, *I* = 11 nA), which is in fact similar to that used in the conventional e-beam-based characterization techniques such as SEM and electron backscatter diffraction, is significantly milder than that required to cause the known e-beam-induced damages such as knock-on atomic displacement and e-beam sputtering^15^.

If the ductility of the Cu film is not improved, how can we explain the crack suppression by e-beam irradiation? The most typical method for suppressing the formation of cracks in a metal thin film on a flexible substrate under tensile stress is to increase the adhesion between the metal film and the substrate^10,11,16^. The main mechanism of cracking in such a film is strain localization arising from its partial delamination from the substrate caused by tensile deformation. Since the delaminated film has the same behavior as a free-standing film, even a slight deformation results in deformation instability, generating cracks due to necking or void formation^17^. This crack formation can be avoided if the delamination of the metal film is prevented by increasing the adhesion. We performed a nanoscratch test on the two Cu  film–PI systems—as before, one unirradiated and the other irradiated. Specifically, the critical normal force (*L*_cr_), which refers to the normal force at the Cu–PI interface at the onset of delamination, was obtained by the nanoscratch test, and the relative value of work of adhesion (*W*) was calculated from the following equation:

1 $$L_{{\mathrm{cr}}} = \frac{{d_{{\mathrm{cr}}}}}{{\nu\mu }}\sqrt {2tEW}$$

where *d*_cr_ is the width of the scratch at the onset of delamination, *ν* is the Poisson ratio of the substrate, *μ* is the friction coefficient at the film–substrate interface, *t* is the thickness of the thin film, and *E* is the Young’s modulus of the substrate^18^. Figure 2d compares the values of *L*_cr_ and relative *W* of the two cases, showing that after e-beam irradiation *L*_cr_ increased from 2.74 ± 0.24 mN to 3.37 ± 0.28 mN, which results in the increase of *W* by a factor of 1.47. This result shows that the suppression of crack formation by e-beam irradiation can be explained by the adhesion enhancement at the Cu–PI interface without an appreciable change in the microstructure of the Cu thin film. The systematic characterization can give clues on the origin of the adhesion enhancement at the Cu−PI interface. Figure 2e shows the electron energy loss spectroscopy (EELS) data collected from different positions at 5 nm intervals across the Cu−PI interface, with the corresponding probe positions denoted on the high-angle annular dark field (HAADF) images. As the probe point moves downward (i.e., toward the PI region), the EELS intensity due to Cu atoms—the peak near 950 eV (the Cu *L*_2_ edge) and the broad signal beyond ~1000 eV—abruptly decreases near the interface for the unirradiated sample (Fig. 2e, left), whereas that of the irradiated sample (Fig. 2e, right) shows a much more gradual decrease across the interface. This result indicates that Cu atoms near the Cu−PI interface in the irradiated sample migrated into the PI substrate, possibly enhancing the interface adhesion by increasing the interfacial area between the Cu and PI regions. Furthermore, the Cu_2_O (111) TEM diffraction pattern observed near the Cu−PI interface (Supplementary Fig. 4) suggests that the interface adhesion may have been further strengthened by the oxide formation between the migrated Cu atoms and the oxygen atoms in the PI substrate, although the Cu_2_O peaks could not be distinguished from the Cu peaks in the EELS data (Fig. 2e) because the EELS signals were not sufficiently strong. The e-beam-induced oxidation of Cu seems plausible, considering our observation based on X-ray photoelectron spectroscopy that the e-beam irradiation caused a chemical change in PI (Supplementary Fig. 5).

### Dependence of crack suppression on the acceleration voltage

**Fig. 3:**
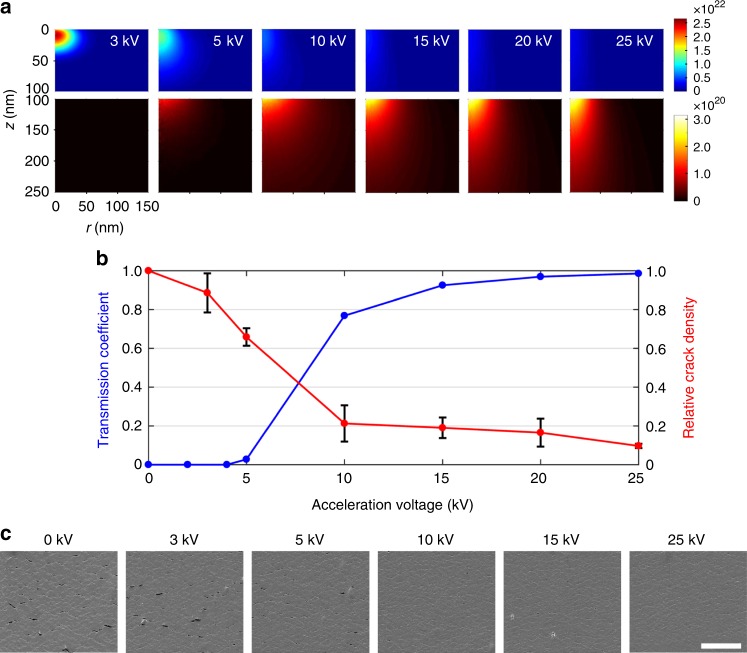
Computational and experimental investigation of the dependence of crack suppression on the acceleration voltage (*V*_A_). **a** Distribution of the energy of the irradiated electrons absorbed in the Cu film–PI system at different values of *V*_A_ ranging from 3 kV to 25 kV. The absorbed energy in the Cu film (0 nm ≤ *z* ≤ 100 nm) and the PI substrate (100 nm ≤ *z* ≤ 250 nm), in unit of keV m^−3^·electron, was calculated using the CASINO Monte Carlo software. **b** Transmission coefficients (defined as the ratio of the number of electrons transmitted through the Cu thin film to that of the total incident electrons) as a function of *V*_A_ obtained from the Monte Carlo calculation (left, blue). Also shown in **b** is the relative crack densities at a tensile strain of 30% (right, red) experimentally determined from the SEM images shown in **c**. Error bars represent the standard deviations. Scale bar, 5 μm

Trajectories of electrons and their interaction with materials, such as elastic and inelastic scattering and energy absorption in materials, can be quantitatively described using a Monte Carlo simulation (CASINO^TM^ software) to understand the electron–matter interactions in this thin film system^19–21^. Figure 3a shows the distribution of the amount of energy absorbed in a 100-nm-thick Cu film and a PI substrate, when an e-beam with a radius of 50 nm is irradiated with different *V*_A_. In each case, the e-beam, consisting of 1,000,000 electrons and centered at (*r*, *z*) = (0 nm, 0 nm), is directed along the positive *z*-direction, and the absorbed energy per unit volume is averaged over the azimuthal angle. When *V*_A_ = 3 kV, the energy of the incident electrons is absorbed entirely in the Cu film. As *V*_A_ increases, the inelastic scattering in the Cu film progressively decreases, resulting in a monotonic decrease of the energy absorbed in that region (upper panels, Fig. 3a). At the same time, the energy absorbed in the PI film (lower panels, Fig. 3a) monotonically increases with *V*_A_. This phenomenon is directly associated with the *V*_A_-dependence of the number of electrons transmitted through the 100-nm-thick Cu film, as shown in Fig. 3b (blue). The ratio of the number of electrons transmitted through the Cu thin film to that of the total incident electrons, starting at zero at *V*_A_ = 3 kV, is only 0.03 at 5 kV, abruptly increases to 0.77 at 10 kV, and reaches a plateau of >0.92 when *V*_A_ ≥ 15 kV, implying that the degree of crack suppression by e-beam is likely to have a strong *V*_A_-dependence.

Hence, we carried out the tensile tests on the Cu  film–PI systems irradiated with *V*_*A*_ varying from 3 to 25 kV, *I* = 11 nA, and D = 4.87 × 10^3^ µC cm^−2^. The SEM images of the Cu thin films obtained at *ε* = 30% shown in Fig. 3c clearly indicate that the crack density in the Cu thin film indeed decreases as *V*_A_ increases. The dependence of crack suppression on *V*_A_ can be more quantitatively seen in Fig. 3b (red), where the crack densities normalized by the crack density of the unirradiated Cu thin film are plotted. The crack densities were determined from the SEM images using an image analysis, the details of which are shown in Supplementary Fig. 6. Figure 3b shows that the degree of crack suppression indeed has a strong *V*_*A*_ dependence, and for a relative crack density < ~0.2, *V*_*A*_ needs to be larger than ~10 kV. This result indicates that the electrons are required to reach the Cu−PI interface with sufficient kinetic energy for effectively suppressing the crack formation, which is consistent with our rationalization that the enhancement of the Cu−PI interface adhesion by the transmitted e-beam is a key mechanism for the crack suppression. E-beam-induced radiolysis of PI, which is strongly suggested by the increases in C–O–C bonds in the irradiated PI substrates (Supplementary Fig. 5) and the increase in indentation hardness of the PI substrate (Supplementary Fig. 7), may have caused the adhesion enhancement: the radiolytic damage could have facilitated both the migration of Cu atoms and the formation of Cu_2_O. Alternatively, the e-beam-induced migration of the Cu atoms may have been caused by the successive momentum transfers from the irradiated electrons to the Cu atoms, analogous to the electromigration^22–24^. Further investigation is required to clarify the mechanism of the adhesion enhancement.

### Application to strain-responsive OLEDs

**Fig. 4:**
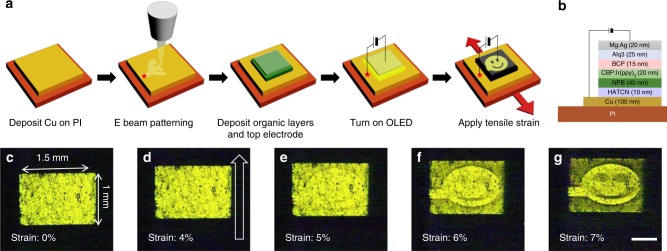
Fabrication and behavior of a strain-responsive OLED patterned by e-beam irradiation. **a** Schematic illustration showing the fabrication and test procedures of the strain-responsive OLED. A red star-shaped dot marked on the Cu film denotes the location of the positive bias. **b** Layer structure of the OLED. **c**–**g** Images of the OLED under a bias of 8 V at *ε* = (**c**) 0, (**d**) 4, (**e**) 5, (**f**) 6, and (**g**) 7%. Red and black arrows in **a** and **d**, respectively, denote the direction of the tensile strain. Scale bar, 500 μm

Our finding can be incorporated into a real device fabrication. Here, we fabricate a strain-responsive optoelectronic device based on the selective formation of a conductivity pattern by the crack suppression technique. The experimental process is schematically described in Fig. 4a. After a Cu layer deposited on a PI substrate was exposed to patterned e-beam irradiation (*V*_A_ = 25 kV, *I* = 45 nA, *D* = 1.00 × 10^4^ µC cm^−2^), small-molecule organic semiconductor layers and a top metal electrode, both being 1.5 × 1 mm in size and covering the patterned region of the Cu layer, were sequentially deposited to form a green phosphorescent OLED with a layer structure shown in Fig. 4b. The e-beam-irradiated area of the Cu layer was in the shape of a smiling face, corresponding to the OLED area that is to remain emissive upon the application of strain, and a DC bias of 8 V, with the positive bias applied on the neck of the smiling face (marked by a red star-shaped dot in Fig. 4a), was maintained during the stretching of the OLED. When *ε*, applied along the direction indicated by red arrows in Fig. 4a, was small (≤4%), the entire (1.5 × 1 mm) OLED was emissive, with small dark spots distributed throughout the device caused by imperfections such as particulates and scratches on the PI substrate (Fig. 4c, d). At *ε* = 5%, the pattern of light-emission intensity corresponding to that of the smiling face began to appear (Fig. 4e) and became increasingly clear as *ε* increased (Fig. 4f, g, in situ video provided in Supplementary Video 2). This intensity pattern is the result of cracks in the Cu layer derived only in the unirradiated area, which increased the electrical resistance of the Cu layer in that region. Consequently, the resistive voltage loss in the unirradiated Cu region was increased, which in turn decreases the values of the local electrical bias and therefore decreases the light intensities in the unirradiated region. This result suggests that with further development, such as the optimized design of the metal–flexible substrate system, the area-selective crack suppression by e-beam may be a versatile technique for patterning various electronic devices in addition to our strain-responsive devices.

In summary, we found that e-beam irradiation onto the surface of a 100-nm-thick Cu thin film deposited on a PI substrate significantly suppresses the crack formation so that the e-beam-irradiated area was nearly crack-free, even at a large tensile strain of 30%, whereas the unirradiated area started crack formation before a strain of 10%. Our experiments and simulations suggested that e-beam irradiation transmitted the Cu thin film and induced the migration of the Cu atoms near the Cu−PI interface, which improved the adhesion at that interface so that crack formation was suppressed during tensile deformation. Since we were able to control the crack formation region in a metallic thin film on a polymer substrate by e-beam patterning, we could also generate any shape of a non-crack pattern in the metallic thin film upon applying a tensile load. These strain-responsive conductivity patterns in a metallic thin film were further incorporated into the fabrication of an OLED, which unveiled a strain-induced light emissive pattern when a tensile load was applied.

## Methods

### Sample preparation

To deposit an array of Cu thin films (thickness: 100 nm, width and length: 100 μm) on a 125-μm-thick PI substrate (Kapton®, DuPont), thermal evaporation using a metallic shadow mask (Supplementary Fig. 1) was employed. A spherically shaped Cu pellet (purity of 99.99%) was used as a deposition source. The base pressure and deposition rate were 2 × 10^−6^ Torr and 8 Å s^−1^, respectively.

### Electron beam irradiation

Prior to e-beam irradiation and SEM observation, 10-nm-thick Pt films were deposited to prevent charging of the non-conducting PI substrate. E-beam irradiation was performed using an SEM (Inspect F or Quanta 3D FEG, FEI). Detailed e-beam conditions are summarized in Supplementary Table 1.

### Fabrication of OLED

The top-emitting OLED has the following structure: PI/100 nm Cu/10 nm 1, 4, 5, 8, 9, 11-hexaazatriphenylene hexacarbonitrile (HAT-CN)/40 nm N,N’-bis(naphthalene-1-yl)-N,N’-bis(phenyl)benzidine (NPB)/20 nm 4,4’-N,N’-dicarbazolebiphenyl (CBP) doped with 3 wt% *fac*-tris(2-phenylpyridine)iridium [Ir(ppy)_3_]/15 nm bathocuproine (BCP)/25 nm tris-(8-hydroxyquinoline) aluminum (Alq_3_)/20 nm Mg:Ag (1:2 mass ratio). All layers were deposited by thermal evaporation in vacuum at a pressure of ~10^−7^ Torr. The deposition rates for all layers were 1 Å s^−1^.

### Characterization

The tensile test was conducted using a microtensile machine (Microtest 200 N, DEBEN) mounted on a specimen holder in the chamber of the SEM, enabling in situ observation during tensile deformation. The Cu thin films on the PI substrate were elongated up to a strain of 30% at a strain rate of 0.05 min^−1^. High-voltage transmission orientation mapping in STEM was performed in an FEI Tecnai^TM^ F20 S/TEM equipped with an ASTAR^TM^ unit. Accelerating voltage, aperture size for the nano-beam diffraction mode, and beam precession angle were 200 kV, 30 μm, and 1°, respectively. EELS spectra, HAADF and high-resolution TEM (HRTEM) images were acquired with a JEOL ARM200 under the accelerating voltage of 200 kV. Fast Fourier transforms (FFT) obtained from the HRTEM images were calculated using Gatan Digital Micrograph^TM^ (DM) software (version 3.5). XPS analysis was performed with a PHI 5000 VersaProbe (ULVAC PHI, Japan) using a monochromatized Al Kα source. The two ends of the OLED sample were fixed on a Microtest 200 N tensile stage in ambient, and both electrodes were electrically connected to a Keithley 2400 SourceMeter® to apply a DC bias of 8 V. Then, the tensile load was applied to the device with a strain rate of 0.05 min^−1^ until the sample was stretched 10% of its original length. A CCD camera (EO-0312c, Edmund Optics) was mounted above the device to record the OLED device performance in real time.

## Supplementary information

Supplementary Information

Description of Additional Supplementary Files

Supplementary Movie 1

Supplementary Movie 2

## Data Availability

All relevant data are available from the authors upon request.
