# Supplementary material for: Selective crack suppression during deformation in metal films on polymer substrates using electron beam irradiation
Source: Nat Commun. 2019 Oct 1;10:4454. doi: 10.1038/s41467-019-12451-8 (PMC6773782; doi:10.1038/s41467-019-12451-8)
Supplement: Supplementary file 1 — Supplementary Information [file 41467_2019_12451_MOESM1_ESM.pdf]

Supplementary Information for

Selective crack suppression in metal films on polymer substrates during deformation using electron beam irradiation

So-Yeon Lee et al.

Supplementary Figures

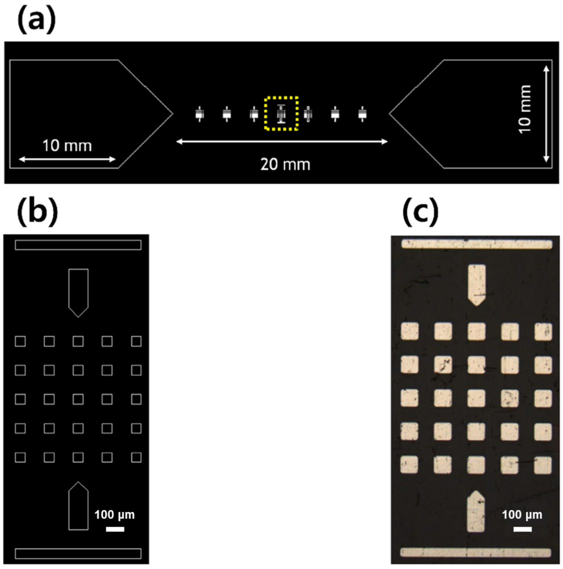

Supplementary Figure 1. (a) The entire shadow mask for patterning the array of Cu thin film pads. (b) The mask image of the array of the Cu pads enlarged from the yellow rectangular area in (a). (c) Optical microscope image of the as-prepared Cu pad array deposited on the PI substrate.

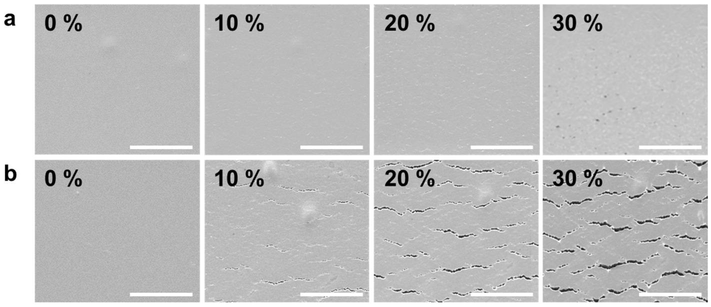

Supplementary Figure 2. Surface morphology of (a) 100-nm-thick Cu film with the electron beam irradiation and (b) 100-nm-thick Cu film with a 10-nm-thick Cr adhesion layer during tensile deformation. The SEM images in (a) are the same as those in Fig. 1c in the main manuscript. Scale bars, 10 μm.

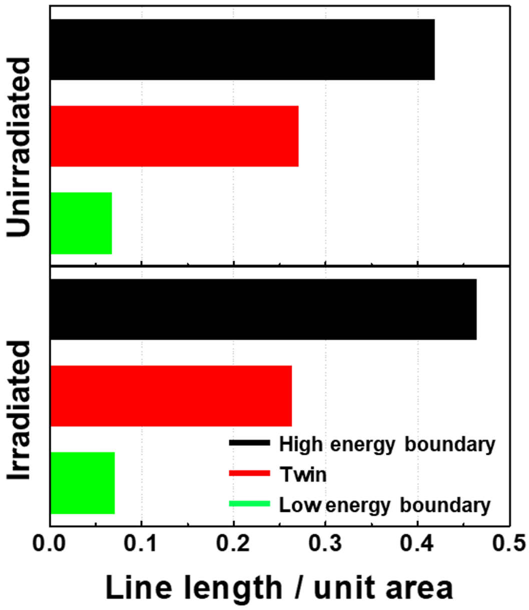

Supplementary Figure 3. Length fractions of the low energy boundary (misorientation angle $< 15°$), the high energy boundary (misorientation angle $> 15°$), and the twin boundary in the unirradiated and the irradiated Cu thin films. The length was normalized to the scanning area of the ASTAR™ analysis ($1\,\mu m \times 1\,\mu m$).

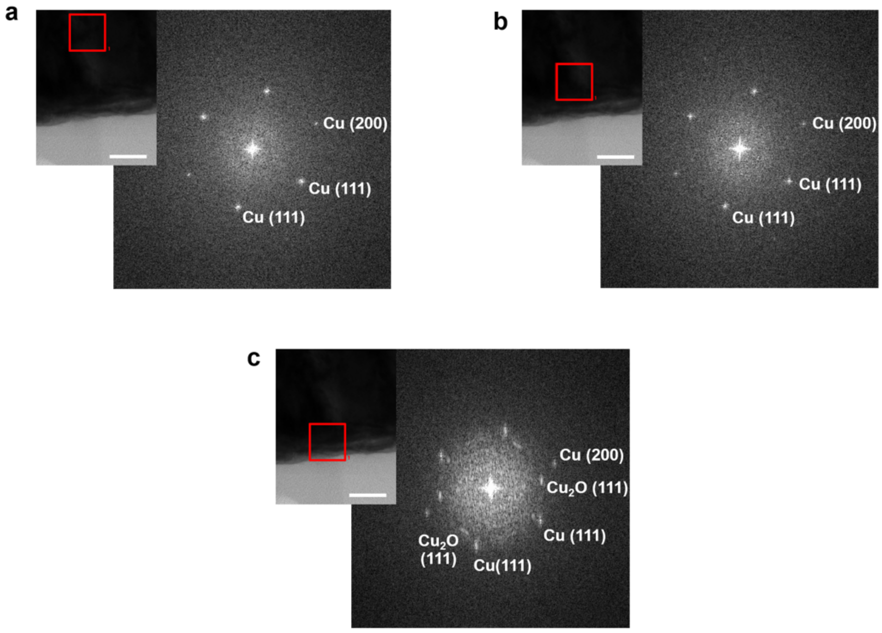

Supplementary Figure 4. (a)–(c) The Fast Fourier Transform (FFT) analysis of the interface regions marked by red rectangles in the cross-sectional TEM images of the irradiated sample. The sample was irradiated with $V_A = 25\,kV$, $I = 11\,nA$, and $D = 4.87 \times 10^3\,\mu C/cm^2$. The analysis shows that the Cu_2O (111) diffraction pattern appears near the Cu–PI interface as shown in (c), suggesting that the interface adhesion may have been further strengthened by additional oxide formation between the migrated Cu atoms and PI by e-beam irradiation. Scale bars, 20 nm.

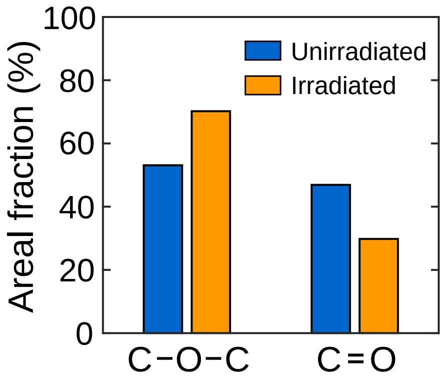

Supplementary Figure 5. We performed X-ray photoelectron spectroscopy (XPS) on two PI substrates without a Cu thin film, one of which was pre-irradiated with electrons. The XPS analysis required that the irradiation areas be much larger than those in the other experiments, which, together with the absence of a Cu layer, significantly increased the degree of electronic charging. It was necessary, therefore, to decrease the values of V_A and D, compared to those in the typical cases (e.g., the experiments corresponding Fig. 1c–e and Fig. 2): $V_A = 5\,\mathrm{kV}$ and $D = 2.84 \times 10^{-2}\,\mu\mathrm{C/cm}^2$. The relative fractions of O atoms participating in the C–O–C and C=O bonds, calculated from decomposing the O 1s XPS spectrum of each case, are shown. The fraction of O atoms forming the C=O bond decreased from 46.9 % to 29.8 %, while the fraction associated with the C–O–C bond increased from 53.1 % to 70.2 %, even with this very mild e-beam irradiation.

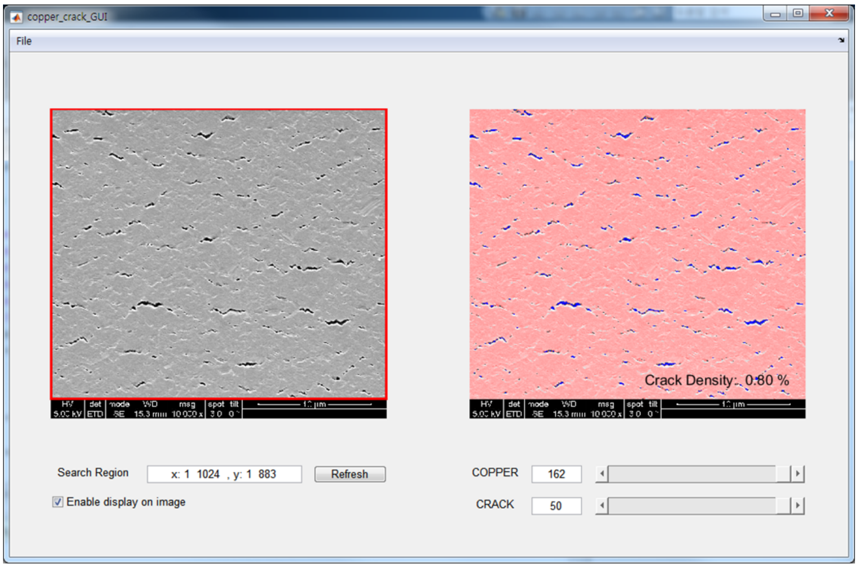

Supplementary Figure 6. Comparing crack density in the Cu thin films. We assumed that the crack density can be estimated by the area fraction of the cracked regions, which was calculated using MATLAB based on the image contrast between the cracked and the uncracked regions.

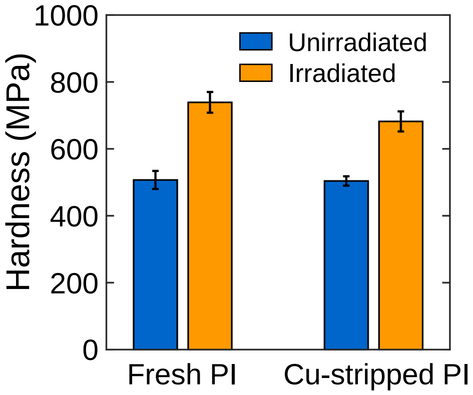

Supplementary Figure 7. Effect of e-beam irradiation on the indentation hardness (H_{I}) of PI substrates. Indentation measurements were first performed on PI substrates onto which a Cu layer had not been deposited (fresh PI) before (blue) and after e-beam irradiation (orange). Next, a 100-nm-thick Cu layer was deposited on another set of fresh PI substrates to form Cu–PI systems, which were then irradiated with an e-beam. After removing the Cu layers by chemical etching, indentation measurements were performed on the resulting PI substrates (Cu-stripped PI) to obtain their H_{I} values (orange). For both fresh and Cu-stripped PI substrates, the e-beam irradiation condition was $V_{\mathrm{A}} = 25\,\mathrm{kV}$, $I = 11\,\mathrm{nA}$, and $D = 4.52 \times 10^4\,\mathrm{\mu C/cm^2}$. Indentation measurements were also performed on Cu-stripped PI substrates that had not been exposed to e-beam irradiation (blue). In the case of the fresh PI substrates, $H_{\mathrm{I}} = (739 \pm 31)\,\mathrm{MPa}$ and $(507 \pm 27)\,\mathrm{MPa}$ for the irradiated and unirradiated cases, respectively. In the case of the Cu-stripped PI substrates, $H_{\mathrm{I}} = (682 \pm 30)\,\mathrm{MPa}$ and $(504 \pm 14)\,\mathrm{MPa}$ for the irradiated and unirradiated cases, respectively, which are similar to the corresponding values of the fresh PI substrates. The e-beam-induced increase in hardness of the Cu-stripped PI substrates is almost comparable with that of the fresh substrates, which suggests that radiolysis occurs in the PI substrates even with the presence of the Cu layer. Error bars represent the standard deviations.

Supplementary Table

Supplementary Table 1. Experimental parameters for e-beam irradiation[a]

	V_A (kV)	I (nA)	Scan time (min)	Horizontal field width (μm)	Dose[b] (μC/cm^2)	SEM model[c]	Spot size setting	WD[d] (mm)
Fig. 1c	25	11	10	130	4.52×10^4	I	6.5	15
Fig. 1d	25	11	10	130	4.52×10^4	I	6.5	15
Fig. 1e	25	11	10	130	4.52×10^4	I	6.5	15
Fig. 1f	25	45	10	746	9.02×10^4	Q	2	10
Fig. 2	25	11	10	130	4.52×10^4	I	6.5	15
Fig. 3	3–25	11	1	125	4.87×10^3	Q	2	10
Fig. 4	25	45	30	4260	1.00×10^4	Q	2	10
Fig. S4	25	11	1	125	4.87×10^3	Q	2	10
Fig. S5	5[f]	0.046[f]	5	7500	2.84×10^{-2}[f]	I	2	15
Fig. S10	25	11	10	130	4.52×10^4	I	6.5	15

[a]For all experiments, the beam dwell time = 300 ns and the screen pixel resolution = 1024×884.
[b]Averaged over the irradiation area.
[c]I = Inspect F; Q = Quanta 3D FEG.
[d]WD = working distance.
[f]The values of these parameters were decreased to prevent the charging of the samples, which became severe due to the increased irradiation areas.
