# Supplementary material for: Selective crack suppression during deformation in metal films on polymer substrates using electron beam irradiation
Source: Nat Commun. 2019 Oct 1;10:4454. doi: 10.1038/s41467-019-12451-8 (PMC6773782; doi:10.1038/s41467-019-12451-8)
Supplement: Supplementary file 2 — Description of Additional Supplementary Files [file 41467_2019_12451_MOESM2_ESM.docx]

Description of Additional Supplementary Files

**Supplementary Movie 1:** In situ observation of the unirradiated and e-beam-irradiated Cu thin film during tensile testing.

**Supplementary Movie 2:** Real-time observation of the strainresponsive OLED
